# Facilitators and Barriers to Implementing Mobile Mental Health Interventions: Qualitative Study of the Consolidated Framework for Implementation Research in Pediatric Oncology Providers

**DOI:** 10.2196/87533

**Published:** 2026-02-23

**Authors:** Shannon J H Hong, Michaela Patton, Krysta S Barton, Tonya M Palermo, Karen Mulholland, Eric J Chow, Nancy Lau

**Affiliations:** 1 Department of Psychology University of California, Berkeley Berkeley, CA United States; 2 Center for Child Health, Behavior and Development Seattle Children’s Research Institute Seattle, WA United States; 3 Stanford Center for Biomedical Ethics Stanford University School of Medicine Stanford, CA United States; 4 Department of Anesthesiology and Pain Medicine University of Washington School of Medicine Seattle, WA United States; 5 Department of Pediatrics University of Washington School of Medicine Seattle, WA United States; 6 Clinical Research and Public Health Sciences Divisions Fred Hutchinson Cancer Center Seattle, WA United States; 7 Department of Psychiatry and Behavioral Sciences University of Washington School of Medicine Seattle, WA United States

**Keywords:** adolescents, young adults, cancer, digital health, mHealth, mental health, mobile phone, psychosocial intervention, implementation science

## Abstract

**Background:**

Adolescent and young adult (AYA) cancer survivors experience unique psychosocial needs during and after treatment. Mobile health (mHealth) interventions are an emerging area of research to help address unmet psychosocial needs. However, few studies have examined provider perspectives on the design-to-implementation pipeline.

**Objective:**

Guided by the Consolidated Framework for Implementation Research (CFIR), our study aimed to examine provider perspectives on facilitators and barriers to implementing mHealth apps in routine clinical care.

**Methods:**

AYA oncology providers participated in a semistructured 1:1 interview on facilitators and barriers to incorporating mHealth apps as psychosocial standard of care. We conducted a directed content analysis of the interviews using a standardized CFIR codebook and construct definitions, with codebook adaptations for mHealth innovations and the population of AYAs with cancer.

**Results:**

A total of 20 providers (mean 39, SD 7.0 years; 80% female and 70% non-Hispanic White) representing various medical and psychosocial roles participated in the interviews. The data were analyzed with 16 CFIR constructs. We identified the following facilitators to mHealth implementation across four CFIR domains: (1) Innovation: alignment with patient needs, patient-centered co-design, strong research evidence, and user-friendly design; (2) Outer Setting: shared commitment to addressing mental health needs and openness to mHealth use; (3) Inner Setting: openness to training on mHealth use; and (4) Individuals: engaging key implementation partners such as bedside nurses and social workers and strong clinical team buy-in. We identified the following barriers to mHealth implementation across three CFIR domains: (1) Innovation: associated costs, (2) Outer Setting: heavy clinical workloads, and (3) Inner Setting: lack of cross-team collaboration and communication and clinical workflow integration.

**Conclusions:**

Our findings highlight key considerations for mHealth co-design, the adoption of mHealth apps into routine care, implementation strategies, and provider training opportunities in the context of AYA cancer care. Partnering with AYA cancer survivors, families, and providers will be crucial for developing and implementing mHealth apps with the ultimate goal of advancing universally accessible evidence-based digital health care.

## Introduction

Approximately 80,000 adolescents and young adults (AYAs) are diagnosed with cancer annually in the United States [1]. Cancer disrupts key developmental milestones for AYAs, such as identity formation, transitioning to independence, relationship development, and achieving educational and vocational goals [2,3]. AYAs living with cancer and AYA cancer survivors (hereafter referred to as AYA cancer survivors) also have unique psychosocial needs that may be unmet throughout their cancer experience [4,5]. Consequently, AYA cancer survivors experience a myriad of mental health issues, including anxiety, depression, and distress, which often persist into long-term survivorship [6,7].

Evidence-based interventions for AYA cancer survivors are a new and emerging area of research [8-11]. The number of AYA-specific psychosocial intervention trials conducted annually has increased significantly from 2 during the years 2007-2014 to 11 during the years 2015-2021 [9]. This growing research activity highlights a commitment to developing evidence-based, developmentally tailored interventions that address the unique needs of this population [12]. As new interventions continue to emerge, leveraging technology has the potential to address barriers that AYAs face in accessing (eg, lack of service availability and financial challenges) and using (eg, time limitations and discomfort in sharing personal problems) face-to-face services [13,14]. As digital natives who have been immersed in technology from a young age, AYAs frequently use digital tools in their daily lives for communication, entertainment, and to access health information and support [15,16]. In recent years, there has been growing research on digital health interventions designed for AYA cancer survivors, with most studies focusing on symptom management, behavior change, self-care, and emotional health [17]. Digital health interventions have demonstrated feasibility and acceptability for AYA cancer survivors [18]. Compared with standard care, digital psychosocial interventions have been found to significantly reduce anxiety and depression and increase resilience in children and AYAs undergoing cancer treatment [10,19]. With 95% of teenagers having access to smartphones [20], mobile health (mHealth) interventions have the potential to expand evidence-based psychosocial care.

On average, it takes 17 years for research evidence to be translated into clinical practice, and only 1 in 5 evidence-based interventions are eventually implemented into routine clinical care [21-23]. While mHealth apps align with AYAs’ digital preferences and hold promise in expanding access to psychosocial care, their integration into clinical care remains a significant research-to-practice gap. Although it is well recognized that successful mHealth implementation depends on the commitment and support of both patients and providers, little prior research has examined provider perspectives [24]. Recent implementation research studies have examined provider perspectives on mHealth tools such as symptom-tracking apps for patients, decision support apps for providers, and cancer triage systems for the general public [25-27]. However, studies have not yet examined provider perspectives on the implementation of psychosocial mHealth interventions. Providers are key stakeholders and partners whose insights are essential for understanding what influences the uptake and implementation of mHealth apps as standard of care within clinical settings.

To address these gaps, we conducted a directed content analysis of qualitative interview data from AYA oncology providers. The primary objectives of our study were to identify facilitators and barriers to implementing new and emerging mHealth apps into routine clinical care from the perspective of AYA oncology providers. Our analysis was guided by the Consolidated Framework for Implementation Research (CFIR), a widely used determinant framework to examine contextual factors influencing implementation effectiveness [28].

## Methods

### Ethical Considerations

This study was approved by the Seattle Children’s Hospital (SCH) Institutional Review Board (number STUDY00004006). All participants provided written informed e-consent via REDCap (Research Electronic Data Capture; Vanderbilt University). Any and all quotes have been deidentified. Providers received a small token of appreciation (ie, water tumbler) for participation in the study. No other monetary incentives were provided.

### Design, Setting, and Participants

Eligible participants were health care providers in the SCH Cancer and Blood Disorders Clinic. All patient-facing providers in the clinic, regardless of role, were eligible to participate in the study. This included psychosocial clinicians (social workers, therapists, and psychologists), oncologists (physicians and fellows), and nurses (nurse practitioners, inpatient, and outpatient) who would potentially integrate mHealth apps into their clinical workflows. Past studies have focused primarily on the perspectives of oncologists. In the interest of inclusivity, we used provider networks across medical and psychosocial staff to try to get equal representation across 6 different patient-facing roles (occupational therapist, pediatric oncologist, psychologist, nurse, social worker, or mental health therapist). We advertised the study on SCH oncology clinic listservs and newsletters and used snowball sampling to identify additional potential participants for recruitment. Participants were queried for convenient appointment times and scheduled for participation in a 1:1 interview. Before the interview, participants completed a brief demographics survey via REDCap.

### Research Team

Authors’ backgrounds included health services research (TMP and NL), implementation science (NL), digital health (SJHH, MP, TMP, and EJC), clinical and pediatric psychology (SJHH, MP, TMP, and NL), pediatric oncology (EJC), psycho-oncology research (MP and NL), and qualitative research (KSB and NL). Authors have extensive background and experience in use of the CFIR framework and qualitative methods (SJHH, MP, TMP, KSB, and NL), which informed study design and research objectives. Authors also have extensive clinical experience with AYA cancer survivors (MP, EJC, and NL), which informed the interview guide, codebook development, and clinical implications drawn from the study.

### Interviews

Semistructured 1:1 interviews were conducted with 20 health care providers via videoconference and were audio-recorded. This sample size was determined based on qualitative research guidelines recommending group sizes of 15-20 to achieve thematic saturation (no new themes or qualifying patterns) [29,30]. Interviews were deidentified and transcribed verbatim. A clinical psychologist trained in qualitative research methods (NL) conducted interviews from July 2024 to September 2024. Individual interviews were 30-50 minutes in length (average 40 minutes) and focused on facilitators and barriers to incorporating emerging mHealth apps as psychosocial standard of care. The interview script is shown in Multimedia Appendix 1. The interviewer memoed during the data collection process. Interviews were conducted at a single cross-sectional timepoint. We adapted a standardized CFIR interview guide for mHealth and the population of AYAs with cancer focused on the CFIR Innovation, Outer Setting, and Inner Setting domains [28].

### Qualitative Analysis

We followed the SRQR (Standards for Reporting Qualitative Research) guidelines for complete reporting of qualitative research data collection and analysis procedures [31]. The completed SRQR checklist is provided in Multimedia Appendix 2. The overall analysis approach was a directed content analysis aimed at identifying facilitators and barriers to implementation of mHealth apps in routine care. We used a standardized CFIR codebook with CFIR domains and constructs, and CFIR definitions. We then developed coding definitions that were specific to mHealth innovations and the population of AYAs with cancer. Using this initial codebook, SJHH, MP, and NL independently coded the first 3 interview transcripts to determine relevance, validity, and fit of each relevant CFIR domain and construct. Over consecutive coding meetings, the analysis team (SJHH, MP, and NL) independently coded transcripts in sets of 3 and iteratively discussed and addressed codebook discrepancies and refined definitions until the codebook was finalized.

The final version of the codebook consisted of 16 of the 67 constructs from the CFIR that were most appropriate to digital mental health interventions. Standardized protocol for applying the CFIR framework is to select a small subset of relevant constructs and domains, specifically operationalized for each research project in accordance with the CFIR qualitative study design guidelines [32]. The most relevant 16 constructs operationalized for our study spanned across all 4 CFIR domains: Innovation (Source, Evidence-Base, Relative Advantage, Adaptability, Design, and Cost), Outer Setting (Local Attitudes and Local Conditions), Inner Setting (Information Technology Infrastructure, Work Infrastructure, Relational Connections, Communications, Recipient Centeredness, Compatibility, and Access to Knowledge and Information), and Individuals (Implementation Team Members). A primary coder (SJHH) and secondary coders (MP and NL) independently coded all transcripts, and all coders met twice monthly to discuss discrepancies and reach consensus. Interview transcripts were managed and analyzed using DeDoose software (SocioCultural Research Consultants).

The interviewer and coder (NL) had prior research relationships with some providers who participated in the study. The interview guide was designed so that questions and probes placed equal emphasis on facilitators and barriers, ensuring that participant feedback covered both domains. Research relationships and solicitations for feedback have historically fostered constructive dialogue, including weighing the pros and cons of different approaches. Dissenting opinions were shared openly and were explicitly encouraged. Reflexive memos and extensive audit trails documenting coding decisions were further used to enhance analytic rigor.

## Results

### Demographics

Participants were 20 oncology providers (mean 39, SD 7.0, range 29-56 years; Table 1). Most of the sample was female (80%) and self-identified as non-Hispanic White (85%). In terms of provider roles, 60% were medical providers and 40% were psychosocial providers. Provider demographics were representative of the overall staff within the clinic. The mean number of years participants had provided clinical care for oncology patients was 10 (SD 9.2, range 1-24) years. On average, participants reported providing care for 20 AYA patients per month (SD 21.0; range 4-100).

**Table 1:** Participant demographic characteristics (N=20).

Participant characteristics		Values, n (%)
**Sex**		
	Female	16 (80)
	Male	4 (20)
**Race**		
	Asian	3 (15)
	White	14 (70)
	Multiracial	3 (15)
**Ethnicity**		
	Hispanic or Latino	1 (5)
	Not Hispanic or Latino	19 (95)
**Role**		
	Occupational therapist	1 (5)
	Pediatric oncologist	8 (40)
	Psychologist	2 (10)
	Nurse (registered nurse or advanced practice provider)	4 (20)
	Mental health therapist	2 (10)
	Social worker	3 (15)

### CFIR Framework

**Figure 1:**
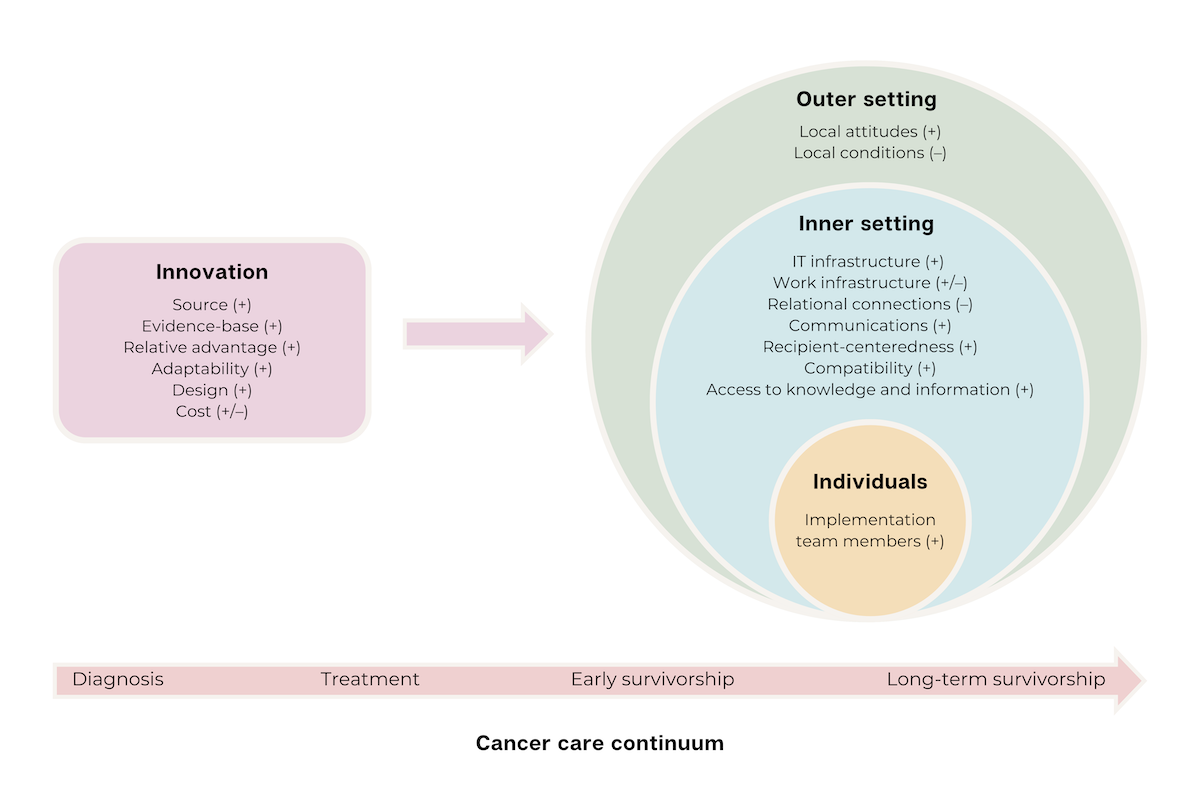
Identified Consolidated Framework for Implementation Research facilitators (+) and barriers (–) to implementing mHealth apps in pediatric oncology settings across the cancer care continuum.

We identified facilitators and barriers to mHealth implementation by using constructs across CFIR domains (Innovation, Outer Setting, Inner Setting, and Individuals) and indicated whether each CFIR construct was a facilitator or barrier using (+) or (–), respectively. Thematic saturation was reached with our study sample. A visual representation of the interrelationships among the Innovation, Outer Setting, Inner Setting, and Individuals domains is shown in Figure 1. We demarcated each CFIR construct as a facilitator or barrier to provide a holistic synthesis of our findings. All findings were relevant to AYA cancer survivors across the cancer care continuum from diagnosis to long-term survivorship. Exemplar quotes for each CFIR construct are shown in Table 2.

**Table 2:** Exemplar quotes of Consolidated Framework for Implementation Research constructs by domain.

Domain and construct		Representative Quotes
**Innovation**		
	Source	“As a professional recommending something to patients, I would want to do better than just generating the type of recommendation patients could find...just Googling. So I would want for someone with some expertise in the area to have evaluated the apps...Good resources would be things [that are] evidence based, sound, scientific foundations, have been used with [an] AYA^a^ cancer population.” [P9]^b^ “I think about programs...that were born out of things that patients were involved in. I think about our AYA, advisory board. People get excited when our own patients are involved in the creation and implementation of things...if patients could take some ownership of this as well and be involved in the beginning stages.” [P14]
	Evidence-base	“I would want a product that was vetted by psychologists and other clinicians and had some sort of proven efficacy...I just want to make sure that anything that I am recommending...is something that is going to provide some sort of medical benefit.” [P1] “A lot of it, of course, is dependent on how engaged the patient is...There are a lot of apps out there that are not evidence based, and so whether, you know, patients are being exposed to concepts or interventions that are anecdotally useful for people but not really evidence based.” [P3]
	Relative advantage	“I think a digital app can be presented and interacted with much more frequently than a face-to-face interaction with a clinician. And you know, because of that, you can get maybe a bit more longitudinal interaction and tracking.” [P1] “We provide really good psychosocial care to our AYA population, which something like this would just enhance. But of the 80,000-ish AYAs that are diagnosed in the US annually...I think something like this app is particularly valuable for reaching those patients who are a sizable proportion of the AYA patient population nationally.” [P9]
	Adaptability	“One of the things that I think is always beneficial are those med reminders [and] symptom management tools where they can document things. Just different tools to help...address that...developmental stage that they’re in and...seeking out more of their independence, autonomy, all those different types of things...plays really deeply into their mental health.” [P7] “It’s very scalable. It would allow AYAs to move through the material at their own pace, according to their own interests on their own time...It potentially could be delivered in a variety of languages with the content potentially tailored to a variety of cultures. So I think it's very scalable, which is important for potential use outside of just our patient population in our institution.” [P9]
	Design	“It’s my clinical experience that [AYAs] like to receive information by video rather than by spoken word or written word. They like content that is short snippets, rapidly paced, attention getting, highly personalized to them in terms of their interests, their issues, their questions...Something that feels more like that is probably going to be more acceptable to them, and so they’d probably be more likely to use it.” [P9] “I feel receptive to an app, especially if it's well designed and relatively intuitive.” [P12]
	Cost	“I would consider cost and what kind of burdens [exist] for signing up. You know, everybody always has to create a username and all this stuff...but I would want a product that was really easy to sign up for and register and wasn't collecting tons of overly invasive stuff or posing extra burdens for an AYA to sign up for.” [P1] “I think the cost and availability of the app would definitely be...in the forefront. If a patient has to commit or get a subscription, I may be more hesitant to recommend that.” [P4]
**Outer setting**		
	Local attitudes	“As I’ve discussed with other nurses and we do see a huge need for mental health support for our patient population and I think that the hospital and the [clinic] program have done a good job of trying to improve upon that and we’ve had more resources and more people join that team.” [P17] “A gap in our psychosocial services that maybe a tool could help us with is transition planning for survivors [with] assessments and then also like resources and interventions for survivors, I don’t think we do a great job.” [P12]
	Local conditions	“Nurses do feel like they’re overburdened with tasks to complete throughout the day and so I do worry that some would and kind of cast it off to the side...It’s just a reminder fatigue, I guess in that sense.” [P17] “One [barrier] is definitely the time and the fact that there's many different providers involved and things can fall through the cracks when they don't feel like the top priority to everyone involved, as there's a lot of things going on.” [P4]
**Inner setting**		
	IT infrastructure	“We have the Get Well Network which is part of our TV system...that included videos...about...what it means to have your counts dropped, what it means to get a central line, all of those things. And alerts pop up on the TV for families to watch those videos...So I’m not sure how you could embed it into that, but I think that could be really helpful to say “reminder, don’t forget to do your [app].” [P17] “Making it a standardized thing in our clinic, whether it’s on the iPads that are in the rooms or...just having the visibility of...the [app] in our clinic, I think would be helpful, especially if it’s proven to be effective. I think having easier access to it, within our clinical space and showing that the clinic backs it up, maybe that would help more patients download it and utilize it at home and take out that a little bit of that stigma piece to it.” [P7] “When you talk to someone about an app, it’s harder to really understand what it looks like or what it entails...[Using TVs] could be a nice opportunity to reach families...while they’re inpatient.” [P18]
	Work infrastructure	“How can there be something that feels...automated and part of the system that then can be sort of supported for patients who have questions about it or who bring it up rather than relying on one person remembering to mention this at a visit or at a different time point.” [P4] “I’m learning a lot more in terms of being a supervisor and how to support patients and family or support my team...when you find the people who have the passion behind the work, things tend to happen more rather than just kind of sitting in this creative process.” [P7]
	Relational connections	“There is a lack of collaboration between our social work and our embedded behavioral and mental health teams. And so, because of that, it’s made a little bit challenging to understand who's doing what and how and where there's reason for overlap...and best practices of how things are delivered and who is delivering it or who’s responsible for it.” [P2] “Factors include, I guess a lack of clarity with differences in clarity with roles and scope of practice. I really think it’s like we’re in such extreme silos, if there is just kind of this often this lack of understanding of what other services do in comparison to your own.” [P14]
	Communications	“Half the battle is gonna be making people aware of [the apps] and buying into their benefit. I think any kind of 1 pager or a few minutes at a meeting, but that both talks about the benefits and the research behind why this is a good approach.” [P5] “The psychosocial group has tried to incorporate...a monthly email that comes out, that’s just a reminder...And then I can refer back to the email and it has bullet points of how to refer and what the criteria are and the dates of the groups. And so something like that could also be helpful.” [P4]
	Recipient centeredness	“I always think about independence and helping [AYAs] have some autonomy in their decision making aspects and or learning the skills so that when they reach the age 18 that it’s not such a shock and preparing them for that transition. So I think helping them create a sense of identity or independence helps their mental health, and especially in this kind of this disease based populations, specifically when they rely so much on their caregivers and their parents or any other adults in their lives.” [P7] “We transition to patients about like a year and a half to two years after therapy to the survivorship for long term follow up team and that might be another point where [the resource] could be helpful...some patients find challenging reintegrating into school or thinking about the fact that scans are upcoming, and each time scans come up that can induce some anxiety. So maybe highlighting some things that other patients or families have cited as stressful and thinking about how these apps could support patients and sort of coping or having increased resilience through that.” [P4] “There’s so much isolation...lack of autonomy, and a lot of issues with self-image that I just would want there to be some sort of other avenue for them to be able to engage in [apps] and be like ‘Hey, you’re not the only person who’s feeling this’ and ‘Here’s some other people that you can talk to about this.’” [P11]
	Compatibility	“For a lot of the patients that I meet with, they’re sort of at the top of the pyramid in terms of their clinical need. Those types of tools could be especially helpful sort of at that universal tier where they’re not necessarily connected to an individual mental health provider, but could get some support that way or do some more kind of self directed skills based learning.” [P8] “In a survivorship clinic visit...it’s been my experience [that] almost universally the first six months off therapy are actually harder than on therapy cancer experience itself. So my thoughts are that almost immediately after that end of therapy visit and scans, that is where...these tools from digital health are potentially most critical and allow for kind of continued way to kind of check in and communicate back. And for the team to get an insight on what's going on such that when patients come back, they...haven’t been suffering from mental health issues and stress and decreased resilience for three to six months by the time they come back to see us for scans.” [P2]
	Access to knowledge and information	“[What] would be the most helpful is if [innovation developers] came probably to one of our meetings and just presented the information to our team. And just kind of let us know what you need from us, and how we can best promote this to the patients. I think that would probably be the first step. I could bring it back to our team and then see if there’s interest, which I’m assuming there would be. And then [innovation developers] could come to our team meeting, and we could talk about it as a as a group about how to go about it.” [P15] “I think better training on [app] functionality and kind of the interventions that they utilize. As well as I think more, kind of research data around their efficacy would be really helpful.” [P7]
**Individuals**		
	Implementation team members	“Social workers for sure often have a really good finger on the pulse and you know, I also think potentially some bedside nursing members or nursing champions on the inpatient unit around that new diagnosis time period could be people who could potentially help in a way.” [P1] “I think people would be incredibly receptive. I know that my nursing colleagues at the bedside would be really excited by something like this. I think that my medical colleagues would also be, you know, social work and psychology, obviously. I think child life is another group who would be very excited. I think a group that we may not sometimes think of as being invested in the mental health of AYAs, but that truly are is PT and OT...we think of them obviously as being involved in promoting physical health, but working with them closely on the inpatient unit, so much of their work focuses on mental health...so much of what PT OT does is really promoting mental health that I think very group who would be very excited by this as well.” [P9]

^a^AYA: adolescent and young adult.

^b^P: provider.

### Innovation

#### Source (+)

Most providers emphasized the importance of mHealth apps being recommended by trusted sources such as colleagues on their clinical team or national medical specialty associations. Providers also highlighted the value of AYA involvement in developing these apps and incorporating their perspectives and feedback, as “people get excited when our own patients are involved in the creation and implementation of [programs and initiatives]” [P14].

#### Evidence-Base (+)

Many providers valued robust scientific evidence demonstrating the effectiveness of mHealth apps and being able to see whether apps would be most beneficial for AYA cancer survivors. One provider stated, “[It would] feel like a wasted opportunity to be able to introduce a useful tool if it’s not grounded in any good evidence and theory” [P3].

#### Relative Advantage (+)

Providers reported several advantages to using mHealth apps compared with traditional clinical practice. Providers emphasized the developmental appropriateness of apps and stated that AYA cancer survivors can “engage within the app in a way that may actually speak their language a bit better than an oncologist sitting in front of them” and that can “facilitate them being able to express themselves in a different kind of way that is probably a little bit more fitting to them” [P3]. Providers also described how mHealth apps could expand access to psychosocial care for AYA cancer survivors following cancer treatment as they transitioned back to a new normal.

#### Adaptability (+)

All providers discussed the adaptability of mHealth apps. Given that AYAs have unique needs and interests, “an app that has really good uptake is [going to be] one that is able to address [what] an AYA is most interested in” [P1]. A common suggestion from providers for tailoring mHealth app content to AYAs’ unique psychosocial needs was incorporating social connection features (eg, connecting with AYA peers with cancer).

#### Design (+)

Providers stated that mHealth apps should be well designed and intuitive to use. One provider stated, “I think it's a great way to get health content...to this population as long as A: the content is sound and B: the delivery is engaging to them. I think those are the two key ingredients” [P9]. Based on their experiences with AYA cancer survivors, providers suggested that content should be presented in an interactive and time-efficient format and be highly personalized to their interests. Some app features of interest include short informational videos, gamification elements (eg, leaderboards and streaks), and mood and symptom monitoring.

#### Cost (+/–)

Nearly half of the sample stated that they were concerned about cost and exclusively interested in recommending mHealth apps that were available for free. Providers emphasized the importance of affordability, with one provider stating, “I wouldn’t recommend ones that are not free, especially if there were a large charge” [P20].

### Outer Setting

#### Local Attitudes (+)

Providers shared a commitment to meeting AYA cancer survivors’ mental health needs and were receptive to using mHealth apps to support clinical needs. One provider stated, “A lot of providers probably...sense the impact of their patients’ mental health on care and may not have the tools...that may be able to help their patients in this way. So [apps] may actually help them feel sort of empowered to at least provide something that the patient could use in the interim before meeting with a mental health provider” [P4].

#### Local Conditions (–)

Some providers identified challenges within the current conditions of the clinic that could hinder the implementation of mHealth apps. A common challenge, particularly among nurses and social workers, was the time constraints in their clinical workload. To address this, providers emphasized the need for systemic support of clinic-wide mHealth implementation efforts. One provider stated, “Some of the buy-in would also have to be systemic support...It would have to come through management to say we want to provide this opportunity for anyone that’s interested to have this time carved out of their clinical day to put this work into it” [P19].

### Inner Setting

#### IT Infrastructure (+)

Many providers recommended integrating information from mHealth apps (eg, data that show that the patient is improving or struggling) into electronic medical record systems. One provider stated, “Incorporating the results or critical information from that tool back into the environment and EMR...is a critical element for the clear communication and for longevity of the app” [P2].

Providers also suggested leveraging existing technological tools in patient rooms, such as iPads and TVs, to support adoption. For example, they described how educational videos and alerts are preloaded onto TVs for patients and their families. Similarly, TVs could be used to display tutorials of the functions and features of mHealth apps and reminders to download and use them.

#### Work Infrastructure (+/–)

Providers highlighted how mHealth apps could help with their limited bandwidth, as “having another tool that doesn’t actually require really additional provider time that we may not have would be super useful” [P3]. However, they emphasized that tasks and responsibilities would need to be integrated or automated into their existing workflow to streamline the adoption of mHealth apps.

#### Relational Connections (–)

Providers noted operating in silos and a lack of awareness about what other clinical teams do and how they collaborate. One provider described having “a lack of clarity...with roles and scope of practice” and “how “we’re in such extreme silos...[that] there is just kind of this lack of understanding of what other services do in comparison to your own” [P14]. As a result, it has been “challenging to understand who’s doing what and how, where there’s reason for overlap...and best practices of how things are delivered and who is delivering it or who’s responsible for it” [P2].

#### Communications (+)

Although there was no consensus about the frequency of sharing information about mHealth apps among providers, they mentioned that a combination of face-to-face and written communication methods, such as email, one-pagers, and presence at team meetings, would be helpful. One provider stated, “Those Friday AM talks...have always been really well-received...I just feel more visibility about these things and the work that we’re doing or that is being done to support our patients from the psychosocial mental health perspective...I feel like the providers are craving it and needing it...And I think it would actually create a lot of buy-in” [P7].

#### Recipient Centeredness (+)

Providers demonstrated shared values around supporting and tailoring mHealth apps to the needs and interests of AYA cancer survivors. A commonly perceived need was social connection. Providers also described how AYAs have previously engaged in gamified activities. For example, one provider shared that AYAs “would get so competitive and see that another teen had gotten a certain number of points and they wanted to try to beat them” [P20]. They suggested that a similar leaderboard feature could be incorporated into mHealth apps to enhance engagement.

#### Compatibility (+)

Providers stated that mHealth apps could “play a role across [the] treatment continuum,” particularly for AYAs getting off treatment and into survivorship as “mental health concerns persist or can absolutely worsen during that time” [P8]. Providers stated that mHealth apps could be “a tool to use especially in moments of distress, which are common in survivorship” or when patients are “coming for scans or labs and have a really high increase of anxiety or stress” [P19]. AYA cancer survivors receiving active treatment also “have a lot of downtime” and mHealth apps would be “a really great way to...harness some of that when they’re between appointments or waiting after labs and before the provider comes in the clinic” [P5]. Providers also described how mHealth apps could be universally used with all AYA cancer survivors, which would “be less of a strain on...the more limited resources that we have for individual patients” [P8].

#### Access to Knowledge and Information (+)

Providers expressed a need for guidance on the benefits of mHealth apps and how to promote them to AYA cancer survivors. One provider stated that “very basic education about...what it’s for and why it’s important” would be needed so that providers “feel engaged and invested” [P4].

### Individuals: Implementation Team Members (+)

Providers recommended bedside nurses and social workers as the clinical team members who would need to be involved as the most ideal potential clinic partners for the successful implementation of mHealth apps. Providers also stated that buy-in and strong engagement from clinical team members would be needed. One provider stated, “You could have created the best thing ever and it won’t make a difference. What you need is a boots-on-the-ground clinic champion to make an impact at the bedside” [P9].

## Discussion

### Principal Findings

To our knowledge, this is the first study to examine facilitators and barriers to implementing mHealth apps in routine clinical care from the perspective of AYA oncology providers. Constructs across the CFIR Innovation, Outer Setting, Inner Setting, and Individuals domains were identified as facilitators and barriers to mHealth implementation. Our study addresses a key gap in the design-to-implementation pipeline of emerging mHealth interventions.

In terms of facilitators to mHealth implementation, a central theme was strong alignment with patient needs. Providers were interested in collaborating on the development of mHealth apps and emphasized that they should be co-designed with AYAs. Strong research evidence and user-friendly design were other key facilitators, with providers stressing that mHealth apps needed to be both effective and engaging for AYA cancer survivors. Providers viewed mental health care as a valuable component of care in oncology and felt optimistic about the potential of mHealth apps to improve patient outcomes and advance mental health equity. They also expressed openness to receiving guidance and training on using and recommending mHealth apps to AYA cancer survivors. Providers identified bedside nurses and social workers as key implementation partners and highlighted the importance of strong clinical team engagement and buy-in.

Providers also identified several implementation barriers. Cost and accessibility were common concerns, with providers expressing hesitancy in recommending subscription-based or high-fee apps. Usability was another common barrier—providers emphasized that AYA cancer survivors would not want to use mHealth apps with a slow user interface. In terms of organizational barriers, providers reported that heavy clinical workloads and limited cross-team collaboration would make it challenging to implement mHealth apps. Providers further acknowledged the potential challenges of integrating these practices into existing clinic workflows, often referencing the high task demands and the time burden associated with supporting mHealth app implementation.

### Comparison With Prior Work

Our findings align with prior research with health care providers who have found positive attitudes and receptivity toward mHealth apps across various health care conditions and disease management functions. A growing body of research also supports the use of mHealth apps in oncology care, which provide opportunities to address patient needs and help them feel engaged throughout treatment [24]. However, to our knowledge, this is the first qualitative study to examine oncology provider perspectives on mHealth implementation. It is also the first to focus specifically on the AYA age group and addressing psychosocial needs in this population. Previous studies that examined provider perspectives have focused on co-designing mHealth interventions with patients and families. Features such as social connection, symptom tracking, and appointment management have emerged as important considerations for these interventions [33,34]. With regard to facilitators of mHealth adoption, our findings align with prior research that has found that providers emphasize the need for evidence-based content and user-friendly design [35]. Our findings support previous recommendations to involve both patients and providers throughout the app development process, ensuring a balance of user and clinical expertise [36,37]. Beyond intervention design, providers offered insights on the integration of mHealth tools and interventions into clinical workflows through electronic health records and existing electronic systems [38]. Providers also shared suggestions for establishing partnerships with boots-on-the-ground clinical champions for large-scale adoptions.

With regard to barriers, our findings align with existing literature on concerns about data privacy, provider liability, and time burden associated with adopting new digital tools [24,34,39]. Our study extends this work by identifying previously unreported barriers, namely, fragmented communication between clinical teams and unclear ownership of implementation tasks. Our findings also highlight opportunities to integrate both medical and psychosocial provider roles into the implementation process and the use of mHealth tools across the cancer care continuum. This includes using mHealth tools during inpatient or outpatient care and throughout the active treatment and posttreatment phases. Additionally, a qualitative review conducted by Jacob et al [40] found that only 3 out of 50 studies examining clinician adoption of mHealth tools used the CFIR framework. This highlights the added value of our study in applying a rigorous implementation science approach to understanding factors that enable and hinder mHealth implementation. While the studies that used the CFIR framework primarily identified structural and organizational factors influencing implementation [41-43], our findings uniquely underscore the value of examining team-level processes when implementing mHealth tools in complex, multidisciplinary care environments.

### Limitations

The findings of our study should be interpreted in the context of several limitations. First, our study was conducted at a single academic hospital, which may limit the generalizability of our results. Providers at this institution may have more experience with or interest in digital health than providers in other clinical settings. Second, our sample lacked demographic diversity, with most participants identifying as female and non-Hispanic White. Although this reflects the provider population in the geographic location in which our study was conducted, it may limit the applicability of our findings to more diverse clinical settings. Third, we intentionally predefined 16 CFIR constructs that were applicable to facilitators and barriers within our settings. With the CFIR having 67 constructs total, future research may explore additional factors that could impact implementation, such as policies, partnerships, or external pressures. Fourth, implementation contexts are dynamic. Our interviews captured the perspectives of providers on mHealth implementation as of 2024.

### Conclusions

Our findings synthesized key implementation considerations for incorporating mHealth apps as a standard of care in psychosocial support for AYA cancer survivors. Based on these findings, recommendations for practice include co-designing mHealth apps with both patients and providers to ensure alignment with user preferences and clinical needs. These tools should then be embedded within existing clinical workflows such as electronic health records to reduce provider burden and ensure seamless integration. Improvements in cross-team collaboration and communication can also be achieved by clearly delineating roles and responsibilities across clinical teams. Additionally, concerns around data privacy, provider liability, usability, and cost should be addressed to support the sustained use and implementation of mHealth apps. Implementation strategies should also include provider training on the benefits of mHealth apps and best practices for recommending these tools to AYA cancer survivors.

Our findings have important clinical implications. Strong partnerships among AYA cancer survivors, families, and providers will be essential for developing mHealth tools that are patient-centered and evidence-based [44]. Importantly, providers of all represented clinical roles believed that mHealth tools had clinical utility and wanted to be involved in the innovation development through implementation pipeline. They were also ready and willing to partner with researchers in early adoption efforts and had suggestions on how to do so. Future research should focus not only on evaluating the effectiveness of mHealth apps but also on implementing strategies that integrate into clinical workflows and minimize provider burden [45,46]. Understanding how these strategies impact provider uptake and patient outcomes will be crucial to moving toward universal evidence-based digital health care using innovative digital health technologies.

## Appendix

Multimedia Appendix 1 Provider interview guide.

Multimedia Appendix 2 SRQR checklist.

## Abbreviations

AYA: adolescent and young adult
CFIR: Consolidated Framework for Implementation Research
mHealth: mobile health
REDCap: Research Electronic Data Capture
SCH: Seattle Children’s Hospital
SRQR: Standards for Reporting Qualitative Research

## Data Availability

The data used in the research cannot be publicly posted and cannot be shared privately with any person because participants did not consent to data sharing. Any shared data, even if deidentified, may result in breach of confidentiality.
