# Supplementary material for: Facilitators and Barriers to Implementing Mobile Mental Health Interventions: Qualitative Study of the Consolidated Framework for Implementation Research in Pediatric Oncology Providers
Source: J Med Internet Res. 2026 Feb 23;28:e87533. doi: 10.2196/87533 (PMC12972683; doi:10.2196/87533)
Supplement: Multimedia Appendix 1 [file jmir_v28i1e87533_app1.docx]

**Multimedia Appendix 1**

**Semistructured interview guide—oncology team**

Thank you for agreeing to participate in this project. Before we begin, I’d like to review a few important points. Please say exactly what you think. Don’t worry about what I think. This is about learning from your perspectives and experiences. This conversation will be recorded so that we can transcribe it and learn from it later. Please do speak up and speak clearly. Any reference to individual people or patients by name will be deleted from transcripts so that no identifiers are left in our research records. That said, identifying information will still remain on the audio file, which will be kept confidential until destroyed. Audio-recordings will be destroyed after we finish analyzing all interview transcripts. Do you have any questions before we begin?

Our goal of these oncology team interviews are three-fold: (1) to understand the unmet mental health needs of your AYA cancer patients, (2) explore your perspectives on mental health or behavioral health apps, and (3) explore how we can incorporate mental health or behavioral health apps into clinical care in pediatric hospital settings. We are collecting both patient and oncology team perspectives. We really value your open and honest feedback.

| (General Potential Probes):  Can you tell me more about that?  Can you fill in some more details? |
| --- |

**Opener:** *From your perspective, what are some of the most important mental health needs of AYAs with cancer?*

**Q1:** *Have you ever recommended mental health or behavioral health apps (e.g., Calm, Headspace) to your patients? What are your opinions about or experiences with mental health or behavioral health apps?*

(Potential Probes):

*(If yes) What did you recommend? How did you decide what to recommend?*

*(If yes) How did you recommend it?*

*(If yes) How did your patients respond?*

*(If no) Have you considered recommending one?*

*(If no) What factors would you consider if you were to recommend one?*

*(If no) What would be appealing or unappealing to your patients about these apps?*

*(If no) What concerns might you have about recommending an app?*

Have you heard of the PRISM program? Promoting Resilience in Stress Management (PRISM) is a resilience coaching program has been shown to improve resilience, distress, and health-related quality of life in AYAs with cancer. It teaches the following coping skills: stress management, goal-setting, cognitive reframing, and meaning-making. As you know, AYAs are often on their phones so we created a mobile-app version of PRISM. The PRISM app is a digital version of the program where patients learn the skills themselves using in-app tutorials and practice opportunities. These next set of questions are about the PRISM app. [Show app screenshots.]

**Q2:** *What are your thoughts about using a mental health app like this with your patients?*

(Potential Probes):

*Tell me about how the PRISM app or something like it would be helpful.*

*Are there some patients apps like this would potentially be more helpful for?*

*How can the PRISM app help complement the care patients already receive?*

*Tell me about how receptive patients would be to the PRISM app.*

*How can the PRISM app help meet the psychosocial needs of your patients?*

*Tell me about how receptive providers like you would be to the PRISM app. What about other types of providers (e.g., oncologist, nurse, social work)?*

*If a patient is already receiving psychosocial treatment with a mental health clinician, would you be more or less likely to recommend this app?*

*What concerns might you have about your patients using an app like this?*

*What other information would you want/need about an app to consider recommending it?*

**Q3:** *How could a mental health app like this fit into your clinic workflows? Please describe in both the inpatient and outpatient settings.*

(Potential Probes):

*How might the PRISM app support your work?*

*How and when could the PRISM app be introduced to patients?*

*What strategies would you recommend to help remind clinic staff to routinely offer the PRISM app to patients?*

*Do you have any recommendations for timing of when patients should receive the app (e.g., during active treatment, survivorship care)?*

*How does the PRISM app align with clinical priorities at the CBDC?*

*Who are the relevant stakeholders for implementing programs like the PRISM app as part of routine care patients receive at Seattle Children’s?*

*Who would be the best advocate(s) embedded in the CBDC to help introduce the PRISM app to patients?*

*How would we get all relevant stakeholders on board to implement programs like the PRISM app in clinic?*

*What factors have helped past implementation initiatives of psychosocial programs to be successful?*

*Are there any other potential facilitators you can anticipate?*

**Q4:** *What are potential barriers to implementing PRISM or other mental health apps in settings like the CBDC?*

(Potential Probes):

*Are there any patients with certain characteristics (such as age, disease, or treatment characteristics) who you think the PRISM app would not be a good fit for?*

*Where might we find some pushback?*

*How might the PRISM app interfere with your work?*

*How might the PRISM app negatively impact the way you do your job?*

*What reasons can you think of that would cause you or other clinicians to be hesitant to incorporate PRISM into patient care?*

*What factors have caused past implementation initiatives of psychosocial programs to be unsuccessful?*

*Are there any other potential barriers you can anticipate?*

**Q5:** *Do you have any other recommendations for how to integrate mental health or behavioral health apps into clinical care?*

**Closer:** *Is there anything else you would like to share that we didn’t cover?*
