# Supplementary material for: Facilitators and Barriers to Implementing Mobile Mental Health Interventions: Qualitative Study of the Consolidated Framework for Implementation Research in Pediatric Oncology Providers
Source: J Med Internet Res. 2026 Feb 23;28:e87533. doi: 10.2196/87533 (PMC12972683; doi:10.2196/87533)
Supplement: Multimedia Appendix 2 [file jmir_v28i1e87533_app2.docx]

**Multimedia Appendix 2**

**SRQR (Standards for Reporting Qualitative Research) checklist**

<http://www.equator-network.org/reporting-guidelines/srqr/>

Checklist from O’Brien BC, Harris IB, Beckman TJ, Reed DA, Cook DA. Standards for Reporting Qualitative Research: A Synthesis of Recommendations. Acad Med 2014 Sept;89(9):1245. doi: 10.1097/ACM.0000000000000388

| **No.** | **Topic** | **Page no(s).** |
| --- | --- | --- |
| **Title and abstract** | | |
| S1 | Title | p. 1 |
| S2 | Abstract | pp. 2-3 |
| **Introduction** | | |
| S3 | Problem formulation | pp. 4-5 |
| S4 | Purpose or research question | pp. 5 |
| **Methods** | | |
| S5 | Qualitative approach and research paradigm | pp. 8 |
| S6 | Research characteristics and reflexivity | p. 6-7, 9 |
| S7 | Context | p. 6 |
| S8 | Sampling strategy | p. 6 |
| S9 | Ethical issues pertaining to human subjects | pp. 6 |
| S10 | Data collection methods | pp. 7 |
| S11 | Data collection instruments and technologies | p. 7 |
| S12 | Units of study | p. 9-10 |
| S13 | Data processing | p. 7 |
| S14 | Data analysis | pp. 7-8 |
| S15 | Techniques to enhance trustworthiness | pp. 8-9 |
| **Results and findings** | | |
| S16 | Synthesis and interpretation | pp. 10-23 |
| S17 | Links to empirical data | pp. 10-23 |
| **Discussion** | | |
| S18 | Integration with prior work, implications, transferability, and contribution(s) to the field | pp. 22-24, 25-26 |
| S19 | Limitations | p. 25 |
| **Other** | | |
| S20 | Conflicts of interest | p. 28 |
| S21 | Funding | p. 27 |
